# Experimental Study on Physicochemical Properties of a Shear Thixotropic Polymer Gel for Lost Circulation Control

**DOI:** 10.3390/gels8040229

**Published:** 2022-04-07

**Authors:** Jingbin Yang, Yingrui Bai, Jinsheng Sun, Kaihe Lv, Jinliang Han, Liyao Dai

**Affiliations:** 1 School of Petroleum Engineering, China University of Petroleum (East China), Qingdao 266580, China; yangjingbin2018@163.com (J.Y.); sunjsdri@cnpc.com.cn (J.S.); lvkaihe@upc.edu.cn (K.L.); hanjlcbm2013@petrochina.com.cn (J.H.); morningstar0506@163.com (L.D.); 2 CNPC Engineering Technology R&D Company Limited, Beijing 102206, China

**Keywords:** polymer gel, shear thixotropic, dilution resistance, high temperature resistance, salt resistance

## Abstract

Polymer gel lost circulation control technology is a common and effective technique to control fractured lost circulation. The performance of a lost circulation control agent is the key to the success of lost circulation control techniques. In this study, rheological tests were used to study the physical and chemical properties of a shear thixotropic polymer gel system, such as anti-dilution, high temperature resistance and high salt resistance. The results showed that the shear thixotropic polymer gel system had the ability of anti-dilution, and the gel could be formed under a mixture of 3 times volume of heavy salt water and 3/7 volume white oil, and could keep the structure and morphology stable. Secondly, the gel formation time of shear thixotropic polymer gel system could be controlled and had good injection performance under the condition of 140 °C and different initiator concentrations. Meanwhile, the shear thixotropic polymer gel system had the ability of high temperature and high salt resistance, and the gel formation effect was good in salt water. When the scanning frequency was 4 Hz and the temperature was 140 °C, the storage modulus (G′) of the gel was 4700 Pa. The gel was dominated by elasticity and had excellent mechanical properties. By scanning electron microscope observation, it was found that the shear thixotropic polymer gel system had a stable three-dimensional reticular space skeleton under the condition of high salt, indicating that it had excellent ability to tolerate high salt. Therefore, the shear thixotropic polymer gel had high temperature and high salt resistance, dilution resistance and good shear responsiveness. It is believed that the results presented in this work are of importance for extending real-life applications of shear thixotropic polymer gel systems.

## 1. Introduction

With the continuous development of oil and gas exploration and development engineering, the geological situation encountered in drilling and development is becoming more and more complex [1,2]. The problem of lost circulation is becoming more and more prominent, especially in fractured formations [3,4,5]. As a major technical problem in the process of oil and gas exploration and development, lost circulation restricts the speed and efficiency of exploration and development [6]. Due to the occurrence of lost circulation accidents, hundreds of millions of economic losses occur every year [7]. Therefore, solving the problem of lost circulation correctly, efficiently and safely has become the research focus of lost circulation control technology at home and abroad in recent years.

The traditional bridging lost circulation materials have some limitations in dealing with the problem of lost circulation [8], and the main limitations are as follows: (a) The compatibility with drilling fluid is poor, and the mixing of lost circulation material and drilling fluid will affect the performance of drilling fluid [9]. (b) The gravity settlement of lost circulation material is severe. It is difficult to fully fill the loss space [10]. (c) Macroscopically, it is easy to cause “false plugging”, and the structural stability of the plugging layer is difficult to guarantee [11]. (d) The shear thixotropic effect is weak, and its ability to retain the plugging layer is not strong [12].

Polymer gel lost circulation materials with the advantages of flexible adjustment, easy injection and adaptive fracture size and shape have gradually become popular in lost circulation materials [13]. Polymer gel is generally composed of a main polymerization agent, crosslinking agent, initiator and a variety of materials, and can achieve different characteristics through the introduction of specific components [14]. In general, polymer gel needs to have certain properties such as temperature resistance and salt tolerance according to different geological conditions [15]. Dai et al. prepared dispersed particle gel (DPG) and studied its macroscopic plugging performance [16]. It was found that when the Young’s modulus of DPG particles increased from 82 Pa to 328 Pa, the plugging efficiency increased from 91.46% to 97.10% [17]. When the Young’s modulus of DPG particles exceeded 328 Pa, the further improvement of plugging efficiency was not significant with the increase in Young’s modulus of DPG particles. However, the shear thixotropic of DPG is poor, and the effect will be greatly reduced after entering the formation. Zhu et al. studied the salt expansion resistance of preformed degradable gel particles (DPPGs) in the laboratory [18]. It was considered that DPPGs showed good swelling ability in a wide salinity range (20,000~400,000 ppm) and could be degraded completely, and the degradation time was prolonged with the increase in temperature [19]. Chen et al. studied the matching relationship between adaptive microgel (SMG) and fractured cores [20]. According to the number and relative pore throat size of SMG, plugging was divided into direct plugging, bridging plugging, superimposed plugging and hydrodynamic plugging. Esfahlan et al. classified different types of PPG and discussed and evaluated the main parameters of gel properties, including particle size, swelling ability, rheological properties and plugging efficiency [21]. Bai et al. used high temperature resistant polymer gel as lost circulation material for drilling [22]. The gel forming time of the gel system was 6~20 h at high temperature. The storage modulus and loss modulus of the gel system were more than 60 Pa and 20 Pa, respectively, which showed good gel strength and viscoelasticity. However, the anti-dilution property and shear thixotropic of the gel are the key to its application. After the gel enters the lost circulation layer and retains the loss space to form the plugging layer, there is the possibility of shear failure under the influence of formation pressure and drilling fluid swabbing pressure [23].

In order to avoid the damage of the lost circulation control layer caused by the influence of pressure, the lost circulation control work requires that the gel should have good toughness to achieve high pressure plugging. Therefore, it is necessary to explore the rheological properties of the gel [24]. Jia et al. developed a high strength and degradable nanocomposite gel for temporary plugging of ultra-low pressure fractured reservoirs, and its initial solution had low viscosity, which proved that the system was easy to inject and had good shear resistance [25]. Meanwhile, the nanocomposite gel system could be gelation in 20–50 min at the temperature of 75–105 °C, which was beneficial to the rapid plugging of fractures [26]. In order to effectively improve the effect of water plugging and oil recovery in fractured reservoirs, Wu et al. prepared a water plugging polymer gel system with long gel time and high strength, and systematically studied the key factors affecting its rheological properties [27]. It was found that excessive crosslinking agent or initiator could make the gel more sensitive to strain, and the critical strain could be reduced to a lower strain amplitude.

The formation of shear-induced gel is due to the seepage transformation of the infinite network structure of anisotropic growth of bentonite particles [28]. On the contrary, the relaxation of the gel was carried out through the isotropic diffusion of bentonite particles [29]. In order to study the reversible shear gelation process of semi-dilute aqueous colloidal dispersions composed of lithium magnesium silicate and weakly adsorbed polymers, Pozzo et al. used the method of small angle neutron scattering and oscillatory shear to find that the shear-induced gelation was time reversible and strongly temperature dependent [30]. In order to improve the shear resistance of amphiphilic polymer gels, Zhou et al. selected cyclodextrin polymer to construct inclusion polymer gels [31], which solved the problem of poor shear resistance of amphiphilic polymer gels. Collini et al. believed that the formation of the gel was affected by shear [32], and the gelation time showed an exponential trend with the increase in shear rate, indicating that the gelation process was related to the kinetic energy and particle motion in the system. Poggi et al. characterized the solid–fluid transition properties of the composite gel under shear induction combined with a continuous shear test and large amplitude oscillatory shear measurement [33]. It was confirmed that the yield strain of the gel becomes smaller at a higher shear rate.

Thixotropic gel is favored by experts and scholars at home and abroad because of its excellent shear properties. In order to plug the fracture with high conductivity and improve the sweep efficiency of remaining oil, Ge et al. used sodium montmorillonite particles prepared in a thixotropic gel system [34]. The thixotropic gel had good rheological properties and was beneficial to the injection and filling of the gel. The viscosity of thixotropic gel could be well matched with the formation at different shear rates, and its shear dilution behavior made the injection pressure of the gel in the injection process lower. Linsha et al. synthesized a rare reversible thixotropic gel by controlling the sol–gel auxiliary reaction of pH [35]. Hydrogel microspheres were prepared by gel granulation technology, which could be used as a new medium to control the interaction between host and guest, especially suitable for adsorption, separation and catalysis. Varadan et al. studied the effect of shear deformation on the static structure factor of gel and considered that shear could affect the long-range structure of gel [36]. At present, the thixotropic gels studied by Ge and Linsha et al. do not involve the gel’s resistance to temperature, salt and dilution. In addition, the control of their gelation time needs to be improved. Therefore, the novelty of this study mainly lies in that the shear thixotropic gel developed has the ability of high temperature resistance, high salt resistance and anti-dilution, and the gelation time is controllable.

The shear thixotropic polymer gel had low viscosity in the flow process and was easy to inject into the formation loss channel. After injection, the viscosity thickens rapidly and easily stayed in the loss channel. After standing, the gel could be cross-linked quickly to form a high strength lost circulation control layer and prevent drilling fluid loss. It had excellent anti-dilution ability and gelation strength, and could slow down the phenomenon of gravity settlement.

Gelation reaction is an important characteristic of shear thixotropic polymer gel systems. From the point of view of physical structure, the shear thixotropic polymer gel system could form cured compounds with certain toughness and three-dimensional reticular structure. Acrylamide is an unsaturated amide that can undergo cross-linking and dehydration. The carbon–carbon double bond could also carry out addition reactions, and has the characteristics of high reaction activity [37,38]. Therefore, acrylamide, which could provide the grafting site of the copolymerization reaction, was selected as the main material of the gel system.

**Figure 1:**
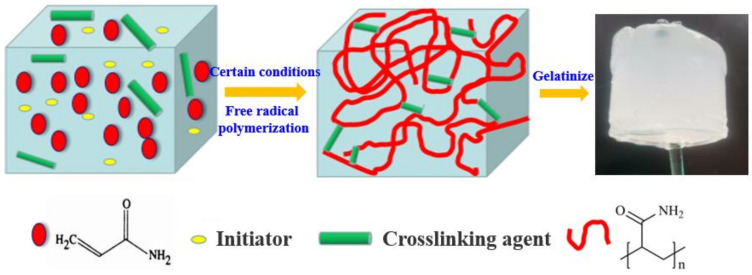
Schematic diagram of gel formation principle of shear thixotropic polymer gel.

From a chemical point of view, the shear thixotropic polymer gel should have a certain degree of crosslinking, and should have temperature resistance in order to prevent high temperature damage to the formation. Therefore, a high temperature resistant crosslinking agent with a large number of carbon–carbon double bond active groups was selected to provide crosslinking sites to increase the crosslinking density [9]. Meanwhile, a certain concentration of initiator was selected to provide free radicals to control the polymerization rate in order to ensure the injection capacity of the gel system. The decomposition rate of initiator carried by the adhesive increased greatly under the action of temperature after it entered the target loss layer. Meanwhile, the increase in the number of free radicals accelerated the polymerization reaction speed [13,39]. A schematic diagram of the gel formation principle of the shear thixotropic polymer gel is shown in Figure 1.

Therefore, the shear thixotropic polymer gel was prepared and its gel properties were studied to improve the shear dilution of the gel to facilitate the injection of the gel. Improving the temperature and salt tolerance of shear thixotropic polymer gel could achieve efficient lost circulation control for different types of fractured formations. This provided an important theoretical and technical support for solving the problem of lost circulation in fractured formations.

## 2. Results and Discussion

### 2.1. Dilution Performance of Shear Thixotropic Polymer Gel

#### 2.1.1. Anti-Dilution Performance of Salt Water

**Figure 2:**
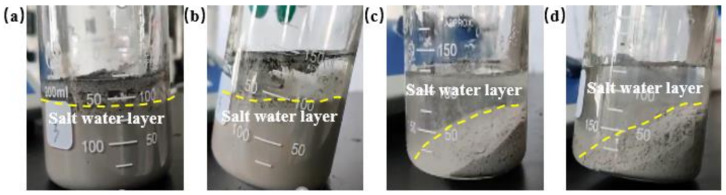
Anti-dilution performance diagram of salt water: (**a**) 1/3 volume salt water; (**b**) 2/3 volume salt water; (**c**) 1/3 volume gel; (**d**) 2/3 volume gel.

“Dilution” refers to the process in which the concentration or structure of the gel decreases due to dilution when it comes into contact with or is mixed with other fluids [40]. In the process of injecting the gel into the loss layer, it was very easily diluted by various fluids and the gelation ability of the gel was affected by the fluid, so the expected lost circulation control effect could not be achieved. Therefore, it was necessary to study the anti-dilution ability of the gel. In order to explore the effect of different fluids on the gelation properties, the experiments were carried out to study the anti-dilution resistance of the gel by replacing deionized water with barite aggravated water and white oil, respectively. In this study, barite was used to increase the clear water density to 1.5 g·cm^−3^ to configure the shear thixotropic polymer gel system, and the shear thixotropic polymer gel system with 1.5 g·cm^−3^ was diluted with high concentration sodium chloride salt water with a concentration of 150,000 mg·L^−1^, and the dispersion state of the gel and salt water was observed. The experimental results are shown in Figure 2.

**Figure 3:**
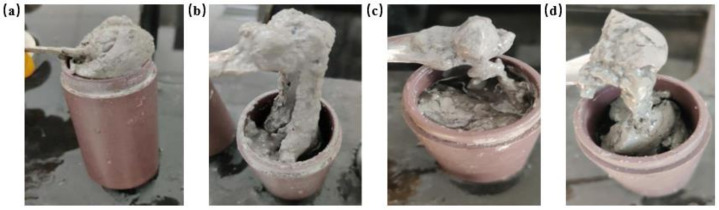
Gelation effect of gel system in salt water: (**a**) 1/3 volume salt water; (**b**) 2/3 volume salt water; (**c**) 1/3 volume gel; (**d**) 2/3 volume gel.

According to Figure 2a,b, it could be found that when salt water was added to the gel system, the morphology of the gel system did not change and the stability was good. Meanwhile, it was found that the gel system accumulated spontaneously in the lower layer of the beaker, and the delamination phenomenon was obvious, which indicated that the gel system could resist the dilution of salt water. Figure 2c and Figure 3d show that the gel was basically not dispersed in salt water, and the gel system was delaminated with salt water. The gel accumulated at the bottom of the beaker and did not mix with salt water, which indicated that the gel system had a good ability to resist dilute salt water.

In addition, the gelation experiment of salt water was carried out on the gel system. The redox initiator was added to the gel system and polymerized at 140 °C for 6 h, and the gelation results are shown in Figure 3. The experimental results showed that the gel could form in salt water after being weighted, but the toughness of the gel system decreased to different degrees. Due to the high quality of salt water in group (c), the gelation effect of the gel system was poor, gel strips appear and the toughness of the gel strips was poor and they were easy to break. The rest of the system could be gelled, and the gel still had a certain toughness after gelation. The experimental results of the four methods showed that the shear thixotropic polymer gel system could resist the dilution of salt water and could gelation when mixed with salt water and maintain good toughness.

#### 2.1.2. Anti-Dilution Performance of White Oil

**Figure 4:**
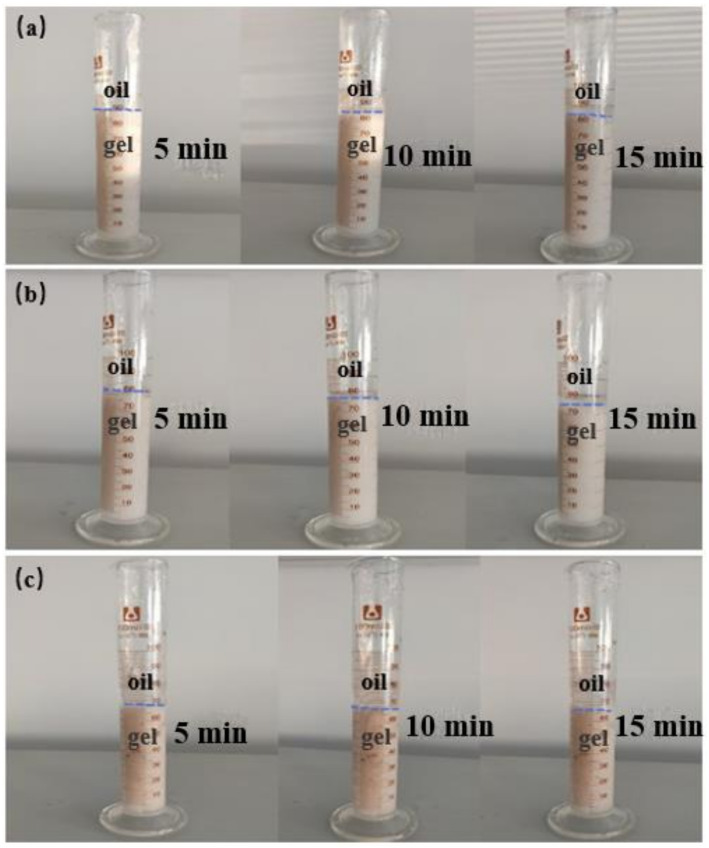
The experimental results of dilute gel system with white oil: (**a**) Gel:white oil = 9:1; (**b**) gel:white oil = 8:2; (**c**) gel:white oil = 7:3.

The anti-dilution performance of gel affected the procedure, use cost and plugging effect of on-site construction operation. The better the anti-dilution performance of the gel was, the less the working procedure was adopted in the construction operation, and the better the lost circulation control effect was [41]. In the experiment, different dilution degrees were set, the gel was mixed with white oil and the dispersion states of gel and white oil were observed under different dilution degrees. Meanwhile, the gelation was tested by the method of mutual dilution between white oil and the gel system, and the gelation in white oil was observed as shown in Figure 4. The experimental results showed that the gel easily accumulated when the volume ratio of the gel system to white oil was 9:1, 8:2 and 7:3, respectively, and the delamination phenomenon began to appear after standing for 5 min, and obvious delamination occurred after 15 min. This showed that there was no interaction between white oil and gel system, which further showed that the anti-dilution effect of the gel system in white oil was good.

**Figure 5:**
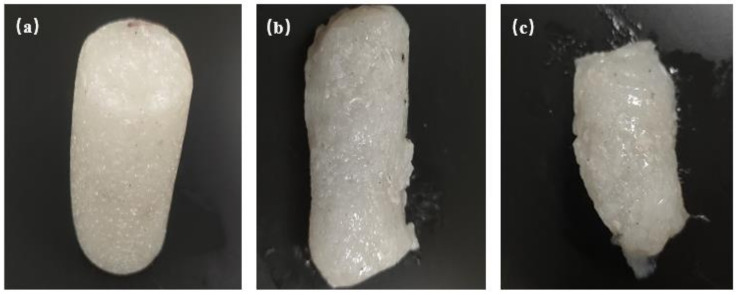
Gelation effect of gel system mixed with white oil: (**a**) Gel:white oil = 9:1; (**b**) gel:white oil = 8:2; (**c**) gel:white oil = 7:3.

The gelation experiments were carried out with the above gel systems with volume ratios of 9:1, 8:2 and 7:3, respectively. The redox initiator was added to the three systems for initiating polymerization at 60 °C for 6 h, and the gelation results are shown in Figure 5. The experimental results showed that the gel system could still achieve good gelation effect when the volume ratio of the gel system to white oil was 9:1, 8:2 and 7:3, respectively, and the gel morphology was intact. The tensile strength of the shear thixotropic polymer gel was tested by a universal testing machine, and the toughness results of the shear thixotropic polymer gel are shown in Table 1. The tensile strength is similar to that of the nanocomposite gel studied by Ning et al. [42]. This showed that the shear thixotropic polymer gel had high toughness and could resist the dilution of white oil. Meanwhile, the three-dimensional network structure of the gel was well formed, and the good toughness was not changed after mixing with white oil.

### 2.2. Gelation Time under Different Initiator Concentrations

The gelation rate directly affected the process of gel migration and plugging in the lost circulation control [22]. On the one hand, when the gelation rate was too fast, migration was difficult. On the other hand, the gel could not reach the deep loss layer, which led to the failure of lost circulation control. The slow gelation rate will also waste the working time and increase the construction cost [43]. Therefore, it was necessary to reasonably control the gelation time of the gel. The gelation time was usually controlled by controlling the concentration of the initiator. The initiator system used in most experiments was ammonium persulfate/sodium bisulfite, and the activation energy was 41.9 kJ·mol^−1^ [44]. In the process of polymerization, not only does a redox reaction takes place, but the activation energy of free radicals was also reduced, so the polymerization could be carried out at a low temperature. However, the decomposition rate of the initiator in high temperature (140 °C) formation was too fast, and the initiation rate was too high, which could easily lead to rapid gelation and increase the difficulty of gel system pumping. Therefore, a hydrogen peroxide tert-butyl alcohol initiator was used to test the gelation time [45]. For this reason, the effect of the gel system on gelation time was studied when the concentration of initiator was 0.1%, 0.2%, 0.3% and 0.5%, and the gel system was placed at 140 °C for 1, 2, 4 and 6 h. The gelation time of shear thixotropic gels under different initiator concentrations is shown in Table 2.

**Figure 6:**
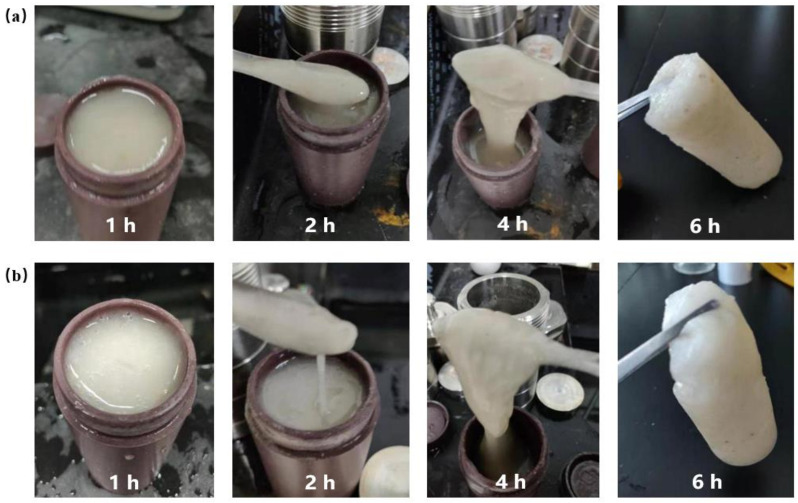
Gel morphology of low concentration initiator at different gelation times: (**a**) 0.1% initiator; (**b**) 0.2% initiator.

Figure 6 showed the gel morphology under different gelation times when using a low concentration initiator. It could be seen from Figure 6a that when the amount of initiator was 0.1%, there were no gel particles or gel strips in the gel solution in the first hour, and the whole gel solution was liquid and not gelatinized. In the second hour, it was found that the gel solution thickened and fine gel particles began to appear, but there was no bulk gel. At the 4th hour, the gel solution became a bulk gel fluid, but the structural strength was low. After standing for 6 h, the gel formed as a whole, showed a certain strength and toughness and the structure was complete. Figure 6b showed the experimental results when the amount of initiator was 0.2%. The gelation rule was similar to that when the amount of initiator was 0.1%, and the gel state began to appear at the 4th hour, but the strength was not high. After reaching the 6th hour, the gel solution gelation as a whole, which had certain strength and toughness and had no flowing liquid. Therefore, the gelation time of the gel system was longer in the condition of a low concentration of initiator. The main reason for this was that the concentration of initiator was too low, too few free radicals were released and the monomer in the gel system was not completely initiated, which led to the gelation time being too long [46]. If the lost circulation occurred in the oilfield, the gelation time was too long, which will waste the lost circulation control time and increase the cost of construction. Therefore, it was necessary to speed up the gelation time of the gel system.

**Figure 7:**
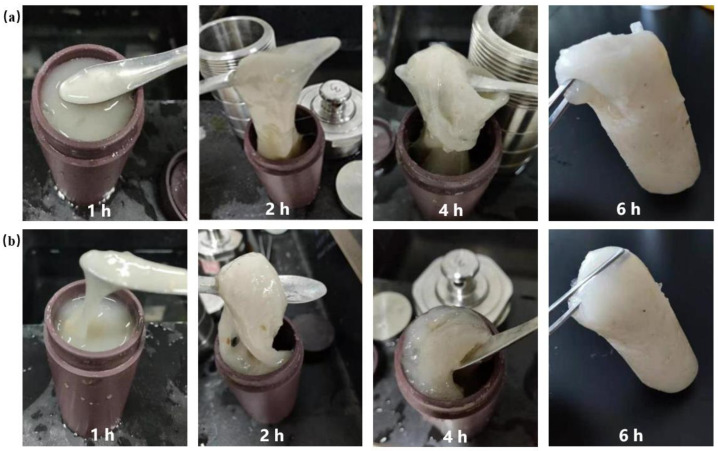
Gel morphology of high concentration initiator at different gelation times: (**a**) 0.3% initiator; (**b**) 0.5% initiator.

Figure 7a showed the experimental results when the concentration of the initiator was 0.3%. Gel particles began to appear in the first hour of the gel system, but the gel particles were tiny and accounted for a very small proportion, and the overall performance was fluid. After the gel system stood for 2 h, large gel strips gradually appeared. At this point, the colloidal volume accounted for about 4/5 of the total gel solution volume. When the gel was stood for 4 h, the gel became solid and had no liquid flow. At the 6th hour, the gel had high strength and toughness. This was mainly due to the free radical polymerization of the gel system in aqueous solution, which emitted heat during the reaction process, and the initiator tert-butyl peroxide was slightly soluble in water. Under the condition of heating, a large number of free radicals were released from the decomposition reaction, which accelerated the gelation time of the gel system. Figure 7b showed the gelation result when the amount of initiator was further increased to 0.5%. The whole gel strip appeared in the gel solution at the first hour, but the structural strength was low. When the gel was stood for 2 h, the gel showed a solid state and lost its fluidity. When the gel was stood for 4 h, the strength and toughness of the solid gel were further improved. When the gel was stood for 6 h, an integral gel with complete structure was formed with high strength and toughness. The experimental results showed that the gelation time of the gel system could be shortened under the condition of high concentration of the initiator. The findings of Zong et al. are similar to those of this study [47], further confirming the validity of this study.

Generally speaking, when the temperature was 140 °C and different initiator concentrations were added, the time required for the gel to change from liquid to solid with certain strength and toughness was different. This was because the weak base groups on the initiator molecules decompose at high temperature, resulting in the breaking of covalent bonds, and free radicals were produced in this process [48]. The production of free radicals could improve the efficiency of the crosslinking reaction and accelerate the polymerization of the system. This promoted the transformation of the gel from liquid to solid. In a certain range, the higher the number of active groups, the faster the polymerization of the system. The gelation time was flexible and adjustable, which further confirmed that the concentration of initiator could affect the gelation time of shear thixotropic gel under high temperature formation conditions.

### 2.3. High Temperature Resistance of Shear Thixotropic Gel

**Figure 8:**
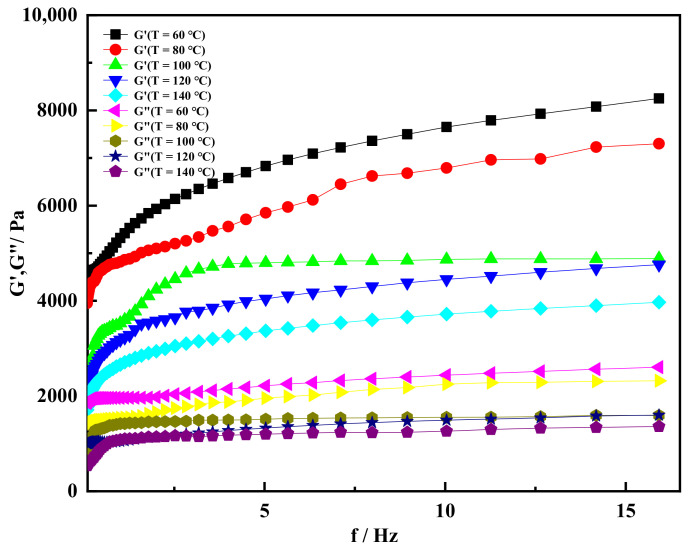
Storage modulus and loss modulus varied with scanning frequency under different temperature conditions.

In order to better study the viscoelastic change trend of shear thixotropic polymer gel at different temperatures, the shear thixotropic polymer gel was placed at 60 °C, 80 °C, 100 °C, 120 °C and 140 °C for seven days to test its rheological properties. Figure 8 showed the variation in storage modulus (G′) and loss modulus (G″) of the gel with scanning frequency at different temperatures. The storage modulus and loss modulus represent the energy stored and consumed when the gel was deformed by external force, respectively [49]. In dynamic rheology, the storage modulus G′ is an important parameter to measure the rigidity of the material, and its value directly reflects the strength of the gel material. The higher the storage modulus, the greater the gel strength [50]. It can be seen from Figure 8 that the storage modulus of shear thixotropic gel increases sharply with the increase in scanning frequency in the range of 0–4 Hz. When the scanning frequency of the gel increased to 4 Hz, the storage modulus of the gel reached the maximum when the temperature was 60 °C, and the storage modulus reached 7000 Pa. When the temperature reached 140 °C, the storage modulus was the lowest, which was 3100 Pa. When the scanning frequency was more than 4 Hz, the increase in storage modulus decreased at different temperatures. When the temperature was 120 °C, the storage modulus of the shear thixotropic polymer gel was almost stable, which was about 4700 Pa. When the scanning frequency reached 16 Hz, the storage modulus of the gel remained stable at all aging temperatures. From the overall trend, the storage modulus of the gel decreased gradually with the increase in temperature, and the elasticity of the gel weakened macroscopically. However, when the aging temperature reached 140 °C and the scanning frequency was 16 Hz, the storage modulus of the gel still reached 3800 Pa, indicating that the thermal stability of the gel was high. The stable three-dimensional network structure maintained a good condition at high temperature.

It can be seen from Figure 8 that the loss modulus increased slowly when the temperature was 60 °C and the scanning frequency was in the range of 0–14 Hz. When the scanning frequency was 16 Hz, the maximum loss modulus of the gel was about 2600 Pa. With the increase in aging temperature, the loss modulus of each gel decreased with the increase in temperature. When the temperature was 140 °C, and the scanning frequency increased to 2 Hz, and the loss modulus of the gel was basically stable, which was lower than that at 60 °C. This was because the intense thermal motion between gel molecules increased the distance between molecules, and the long chains of some gel molecules began to unwrap, which led to the decrease in viscosity macroscopically [51]. Meanwhile, the storage modulus of the gel was always greater than the loss modulus of the gel on the basis of the same temperature and the same scanning frequency. This indicated that the gel was a solid gel dominated by elasticity.

**Figure 9:**
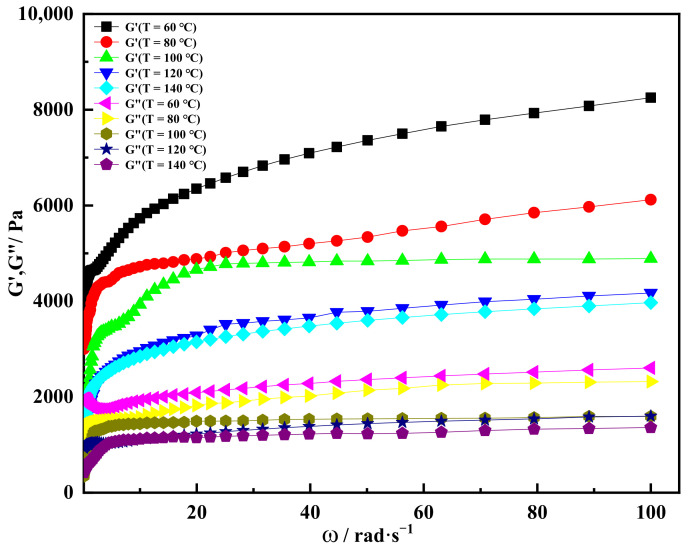
Storage modulus and loss modulus varied with angular frequency under different temperature conditions.

In order to further study variation in storage modulus and loss modulus of the shear thixotropic polymer gel at different temperatures, the angular frequency change scanning test was carried out. It can be seen from Figure 9 that the storage modulus of the gel increased sharply at first and then increased slowly with the increase in angular frequency. When the angular frequency was 40 rad·s^−1^, the storage modulus of the shear thixotropic polymer gel decreased with the increase in temperature. When the temperature was 60 °C, the storage modulus of the gel was about 7200 Pa. When the temperature reached 140 °C, the gel still had a high storage modulus of 3800 Pa. Meanwhile, when the temperature was 60 °C and the angular frequency was in the range of 20–100 rad·s^−1^, the loss modulus of the gel increased slowly, and the increase amplitude decreased gradually. When the angular frequency was 100 rad·s^−1^, the maximum loss modulus of the gel was about 2600 Pa. When the temperature was 140 °C, the loss modulus of the gel was the lowest at the same angular frequency. In addition, it could be found that the storage modulus of the gel was larger than the loss modulus at the same temperature and angular frequency, indicating that the gel was dominated by elasticity and had high structural strength and good mechanical performance at high temperature.

**Figure 10:**
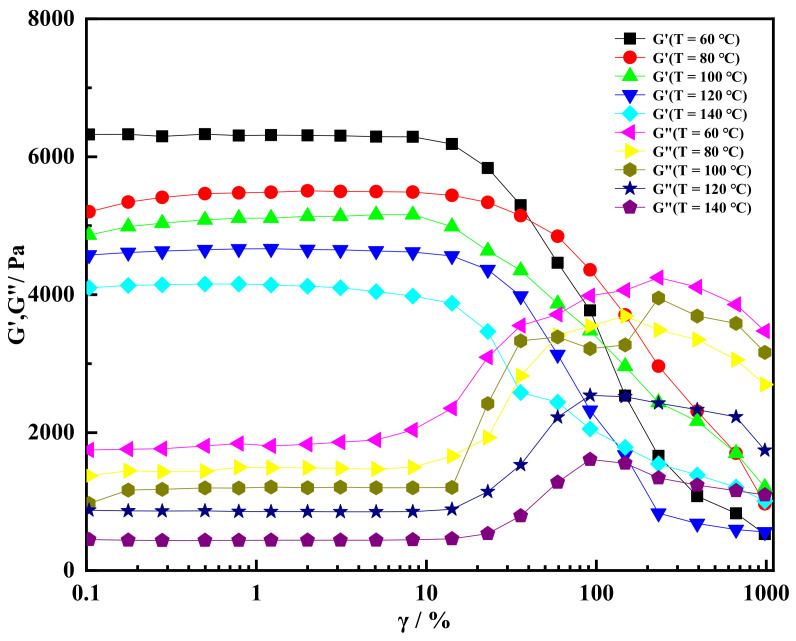
Storage modulus and loss modulus varied with strain at different temperatures.

The storage modulus and loss modulus of shear thixotropic gel at different temperatures were studied by strain scanning experiment. According to Figure 10, the storage modulus of shear thixotropic gel tended to be stable first and then decrease gradually with the increase in strain. On the contrary, the loss modulus stabilized first and then increased with the increase in strain. Meanwhile, the storage modulus and loss modulus of shear thixotropic gel gradually decreased with the increase in temperature. When the temperature reached 140 °C and the strain was 10%, the storage modulus of shear thixotropic gel could still reach 4100 Pa, indicating that shear thixotropic gel still had good structural strength at high temperature. This research result was consistent with the study of Lipatova and Ningtyas [52,53], which further proves the accuracy of the research result.

**Figure 11:**
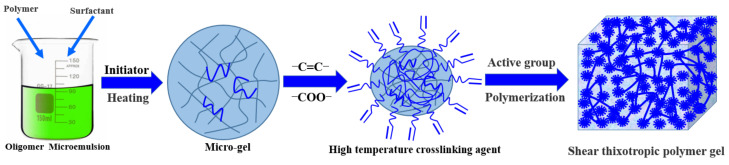
High temperature resistance mechanism of shear thixotropic polymer gel.

In summary, the shear thixotropic polymer gel still had a high storage modulus at high temperature, indicating that the shear thixotropic polymer gel had good high temperature resistance. This was mainly due to the high temperature resistant crosslinking agent synthesized by reverse micro-emulsion polymerization in the shear thixotropic polymer gel system. There were a large number of unsaturated carbon–carbon double bonds and unsaturated functional groups in the molecular chain of the high temperature resistant crosslinking agent [9]. It was easily copolymerized with other monomers with crosslinking sites to form a stable three-dimensional network structure (Figure 11). The crosslinking agent had relatively large molecular weight and long molecular chain and had more and longer side groups. The existence of a long side chain could protect the main chain and effectively control the degree of freedom of the rotation of the main chain. Due to the influence of steric hindrance after polymerization, it took a lot of energy to make the molecules of the polymerization products break, and the thermal stability of the polymerization products was good [54]. Meanwhile, the shear thixotropic polymer gel had more crosslinking sites, which could increase the crosslinking density of the polymerization products to a certain extent. The crosslinking mode was carbon–carbon crosslinking with a strong stable structure, which could improve the temperature resistance of the gel.

### 2.4. High Salt Resistance of Shear Thixotropic Polymer Gel

#### 2.4.1. Effect of Salt Concentration on Gelation

**Figure 12:**
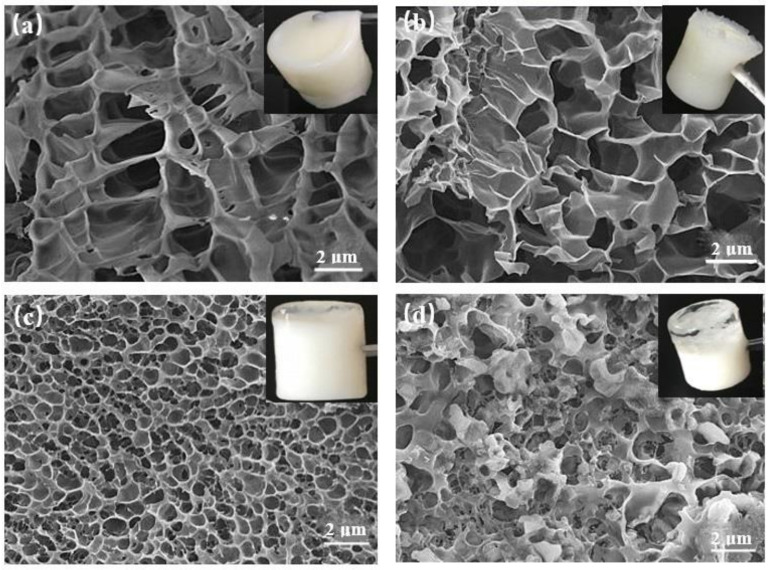
Micromorphology of shear thixotropic gel in different salt water concentrations: (**a**) 10,000 mg/L NaCl; (**b**) 100,000 mg/L NaCl; (**c**) 1000 mg/L CaCl_2_; (**d**) 10,000 mg/L CaCl_2_.

The salt resistance of the gel system was an important index to evaluate its performance. The salt ion solutions of different valence states and concentration gradients were used to replace the deionized water, and the gelation experiments were carried out under the same initiation conditions, and the morphological and structural stability of the gel were observed. The experimental results are shown in Figure 12. The experimental results showed that the shear thixotropic polymer gel system could form gel under the condition of a high concentration of sodium chloride and calcium chloride, and the gel toughness was strong. By observing the micromorphology of shear responsive gel using a scanning electron microscope, it could be found that the shear responsive gel could still maintain a good three-dimensional reticular skeleton structure under the condition of a high concentration of salt water. This was mainly due to the fact that the shear thixotropic polymer gel system was based on acrylamide and other structural units with special functions were introduced through polymerization modification and copolymerization to synthesize polymers with temperature resistance and salt resistance [55]. It could copolymerize with other monomers with crosslinking sites to form a stable three-dimensional network structure. Meanwhile, the crosslinking agent provided enough crosslinking sites to make the network structure of the polymerization products more compact and stronger.

#### 2.4.2. Effect of Univalent Salt Ion Concentration on Gelation

**Figure 13:**
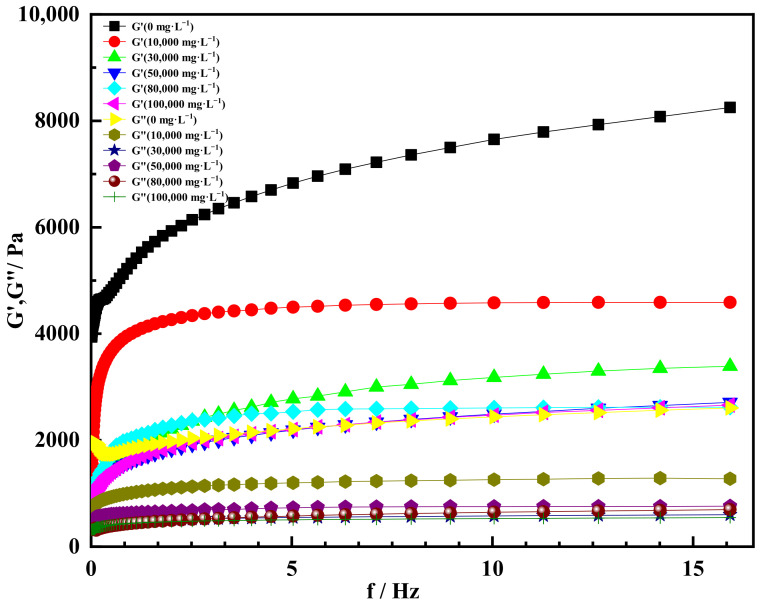
Storage modulus and loss modulus varied with scanning frequency at different concentrations of univalent salt ions.

The gel solution was prepared by simulating formation water with different univalent salt ion concentration solutions, and the gel solution was placed at a constant temperature (60 °C). The rheological properties were tested after it was completely solidified. The ion concentrations of univalent salt simulated by NaCl solution were 0, 10,000, 30,000, 50,000, 80,000 and 100,000 mg/L, respectively. The experimental results are shown in Figure 13. It could be seen that under the condition of different univalent salt ion concentrations, the storage modulus of the gel increased with the increase in scanning frequency, and finally tended to be stable. When salt water was not added and the scanning frequency was 2 Hz, the storage modulus of the gel was 5700 Pa. When the scanning frequency was 16 Hz, the storage modulus of the gel increased to 8200 Pa. This was due to the fact that the stress frequency of the gel increased, and the deformation site was not fully recovered, showing an increase in storage modulus. However, the deformation of the gel basically did not change with the further increase in scanning frequency, so the macroscopic elasticity basically did not increase. In addition, it could be found from the curve that the storage modulus of the gel decreased with the increase in the concentration of univalent salt ions. The main reason was that the presence of salt ions could compress the stretch state of gel molecules and affect the strength of the gel’s three-dimensional network structure [56].

In addition, the loss modulus of the gel decreased at the same scanning frequency with the increase in the concentration of univalent salt ions. The reason was that salt ions in inorganic salts hinder the extension of gel molecules. The higher the concentration of inorganic salts in the deformation process, the more obvious the hindrance effect of metal salt ions on the extension of gel molecular chains, thus reducing the loss modulus of the gel [57]. Meanwhile, it could be seen that the storage modulus of the gel was much larger than the loss modulus, indicating that the elasticity of the gel was dominant. The gel had the characteristics of typical solid elastomers, and the gelation ability was good under the condition of univalent salt ion concentration.

**Figure 14:**
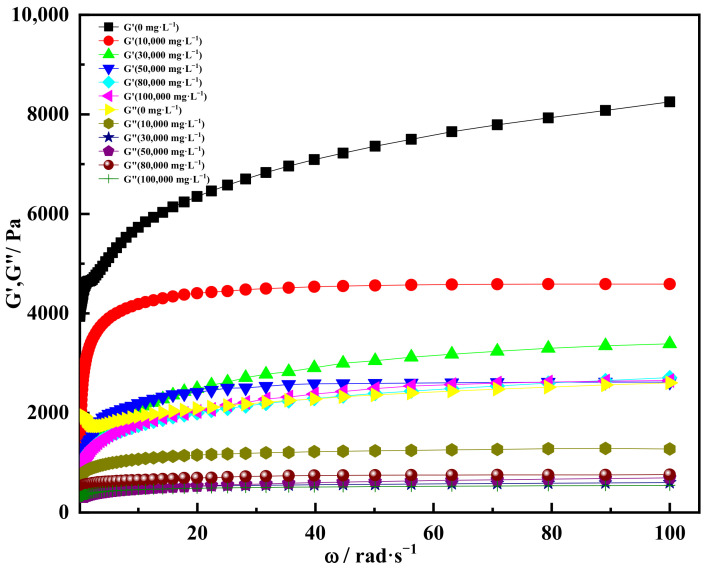
Storage modulus and loss modulus varied with angular frequency at different univalent salt ion concentrations.

Figure 14 showed that the curve of storage modulus and loss modulus varied with angular frequency at different univalent salt ion concentrations. It could be found from Figure 14 that the storage modulus of the gel increased at first and then tended to be stable with the increase in angular frequency under the condition of different univalent salt ion concentrations. When the concentration of univalent salt ions was 0 and the angular frequency was 100 rad·s^−1^, the storage modulus of the gel was 8100 Pa. At the same angular frequency, when the concentration of univalent salt ions was 10,000 mg/L, the storage modulus of the gel was 4200 Pa. When the univalent salt ion concentrations were 30,000, 50,000, 80,000 and 100,000 mg/L, the storage modulus of the gel tended to be consistent and stabilized at about 2500 Pa, indicating that the gel could still maintain good elasticity and the gel structure was stable.

When the concentration of univalent salt ion was 10,000 mg·L^−1^ and the angular frequency was in the range of 0.1–20 rad·s^−1^, the loss modulus of the gel increased sharply at first and then tended to be stable. When the angular frequency increased to 40 rad·s^−1^, the loss modulus of the gel remained stable at 1350Pa. When the univalent salt ion concentrations were 30,000, 50,000, 80,000 and 100,000 mg/L, the loss modulus of the gel was almost the same, about 500–750 Pa, indicating that the univalent salt ion concentration had little effect on the loss modulus of the gel. The main reason was that the gel crosslinking agent used contained more crosslinking sites, which increased the crosslinking density of the gel, and the three-dimensional network structure of the gel was stable and the structural force was strong [58]. Meanwhile, the crosslinking site weakened the blocking effect of inorganic metal salt ions on the molecular chain, and the loss modulus changed little [59]. It can be found from Figure 14 that the storage modulus of the gel was larger than the loss modulus at the same angular frequency, indicating that the gel was dominated by elasticity and had a good gelation effect under the condition of univalent salt ion concentration.

#### 2.4.3. Effect of Divalent Salt Ion Concentration on Gelation

**Figure 15:**
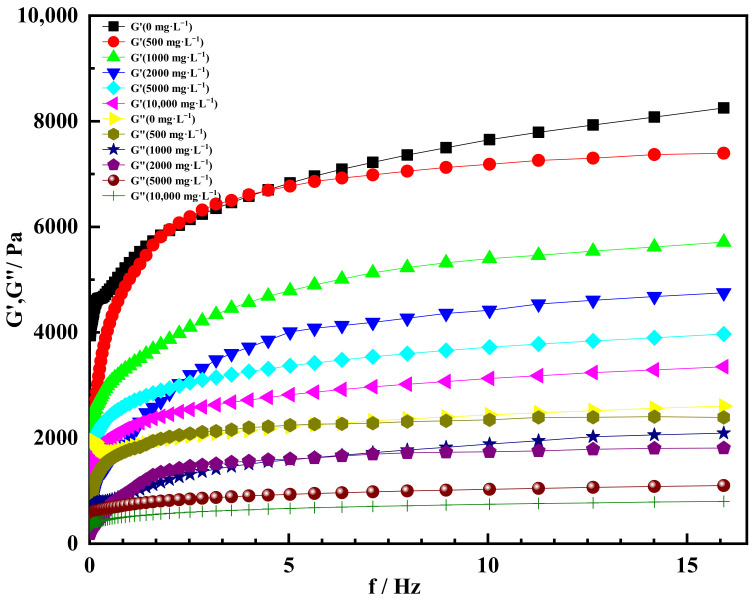
Storage modulus and loss modulus varied with scanning frequency at different concentrations of divalent salt ions.

**Figure 16:**
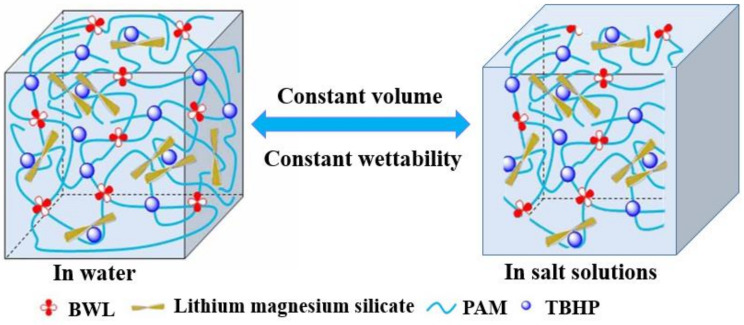
Schematic diagram of partial structural collapse of shear thixotropic polymer gel.

The gel solution was prepared by adding different concentrations of divalent salt ions to the simulated formation water and placing it at a constant temperature of 60 °C. After it was solidified, the change in its rheological property was tested. The divalent salt ion concentrations simulated by CaCl_2_ solution were 500, 1000, 2000, 5000 and 10,000 mg/L, respectively. The experimental results were shown in Figure 15. It could be seen from Figure 16 that the storage modulus of the gel increased with the increase in scanning frequency under the condition of divalent salt ion concentration. When the scanning frequency was 0–2 Hz, the increasing amplitude increased sharply, but when the scanning frequency was more than 2 Hz, the increasing amplitude of the storage modulus decreased gradually and remained almost unchanged. When the scanning frequency was 16 Hz, the storage modulus of the gel with a divalent salt ion concentration of 0 was 8100 Pa. At the same scanning frequency, the storage modulus of the gel with a divalent salt ion concentration of 500 mg/L was about 7200 Pa. When the scanning frequency was the same, the overall storage modulus of the gel decreased with the increase in the concentration of divalent salt ions. When the concentration of divalent salt ions was 10,000 mg/L, the storage modulus was about 3200 Pa. The decrease in the storage modulus of the gel was mainly due to the decrease in the elasticity of the gel, and the valence and concentration of metal salt ions in inorganic salts affected the extension of the gel molecular chain. The higher the concentration and valence of metal salt ions, the easier it was to compress the extended state of the gel molecular chain. Overall, the storage modulus of the gel was still above 3000 Pa under the condition of different divalent salt ion concentrations, indicating that the gel could still maintain a good gelation effect under the condition of high divalent salt ion concentrations.

The loss modulus of different divalent salt ion concentrations increased at first and then tended to be stable with the increase in scanning frequency. When the scanning frequency was more than 2 Hz, the increased amplitude of loss modulus decreased gradually, and the trend line was almost a horizontal line. When the concentration of divalent salt ions was 500 mg/L and the scanning frequency was 16 Hz, the loss modulus of the gel was about 2500 Pa. When the concentration of divalent salt ions was 10,000 mg/L and the scanning frequency was 16 Hz, the scanning frequency of the gel was about 800 Pa. It could be seen from the curve that the loss modulus decreased with the increase in the concentration of divalent salt ions. This indicated that the shear thixotropic polymer gel was enhanced by the shielding charge of inorganic salts with the increase in the concentration of inorganic metal ions [50]. The molecular chain of the gel partially curled, and the three-dimensional network structure of the gel partially collapsed (Figure 16) [60]. In the process of increasing the concentration of divalent salt ions, the storage modulus was always larger than the loss modulus, indicating that the gel had high solid strength and could withstand a high divalent salt ion environment.

**Figure 17:**
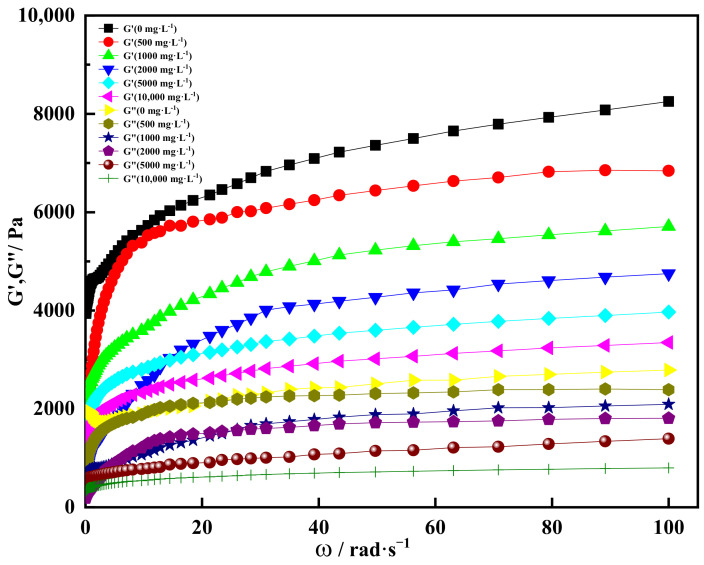
Storage modulus and loss modulus varied with angular frequency at different concentrations of divalent salt ions.

Figure 17 showed the curve of storage modulus and loss modulus with angular frequency at different concentrations of divalent salt ions. It could be seen from Figure 17 that the storage modulus of the gel increased with the increase in angular frequency under the condition of different divalent salt ion concentrations. When the angular frequency was more than 10 rad·s^−1^, the storage modulus increased with the increase in angular frequency. When the angular frequency reached 100 rad·s^−1^ and the concentration of divalent salt ions was 0, the storage modulus of the gel was 8100 Pa. At the same angular frequency, the storage modulus of the gel was 7300 Pa when the concentration of divalent salt ions was 500 mg/L. When the concentration of divalent salt ions increased to 10,000 mg/L, the storage modulus of the gel decreased to about 3200 Pa. This indicated that the concentration of heavy metal ions in inorganic salts affected the extension of the gel molecular chain and reduced the storage modulus of the shear thixotropic polymer gel, thus weakening its elasticity [61]. In the whole range, the storage modulus of the gel could be kept above 3000 Pa under the condition of different divalent salt ion concentrations, which had good mechanical properties.

Meanwhile, the loss modulus of shear thixotropic polymer gel increased with the increase in angular frequency. When the angular frequency was more than 20 rad·s^−1^, the increase amplitude of loss modulus decreased gradually, and the trend line was close to a horizontal straight line. When the angular frequency was 100 rad·s^−1^ and the divalent salt ion concentration was 500 mg·L^−1^, the loss modulus of the gel was about 2350 Pa. Under the same angular frequency, the loss modulus of the gel with a divalent salt ion concentration of 10,000 mg·L^−1^ was 700 Pa. In general, the loss modulus of the gel decreased with the increase in divalent salt ions, indicating that the viscosity of the gel was also decreased. In addition, it could be seen that the gel storage modulus with different divalent salt ion concentrations was always greater than the loss modulus. This showed that the gel had a typical solid structure, and the gel was dominated by elasticity. The gel could be formed under the condition of divalent salt ions.

In summary, the gel system could gelation under the conditions of different concentrations and different valence states of salt ions. In general, the storage modulus of the gel decreased with the increase in salt ion concentration and valence, and the loss modulus decreased with the increase in salt ion concentration. The reason was that the shear thixotropic gel was enhanced by the shielding charge of inorganic salts, and the functional groups of gel molecules will have a strong interaction with salt ions, so the gel network structure became compact. Meanwhile, salt ions easily formed calcium carboxylate, which shortened the gel molecular chain and reduced the three-dimensional network volume of the gel, which further hindered the formation of a spatial physical crosslinking network structure of the gel and reduced the strength of the gel macroscopically.

## 3. Conclusions

Shear thixotropic gels have the characteristics of self-adapting to the loss space, and their adaptability to complex geological conditions is the key to forming a good lost circulation control layer. In order to study the lost circulation control adaptability of shear thixotropic gels in a complex formation, the gel system’s dilution resistance, high temperature resistance and high salt resistance were evaluated, and the effects of temperature and salt ion concentration on gelation performance were studied by rheological analysis. The research conclusions are as follows:

(1) The shear thixotropic polymer gel system had the ability of anti-dilution. The gel could be formed under the condition of mixing 3 times volume of heavy salt water and 3/7 times volume of white oil, and it could maintain the stability of structure and morphology. Meanwhile, the gelation time of the shear thixotropic polymer gel system could be controlled in 2–6 h under the condition of 140 °C and different initiator concentrations.
(2) The shear thixotropic polymer gel had the ability to resist high temperature, and the storage modulus of the gel was greater than the loss modulus at 140 °C When the temperature was 140 °C and the scanning frequency was 16 Hz, the storage modulus of the gel still reached 3800 Pa. Shear thixotropic gels could maintain a good three-dimensional network structure at high temperature and have certain high temperature stability.
(3) The shear thixotropic polymer gel system had stable three-dimensional reticular structure and excellent salt resistance under the condition of high salt. It was found that the gel had good gelation effect and strong resistance to high salt in 100,000 mg/L sodium chloride and 10,000 mg/L calcium chloride. When the scanning frequency was 4 Hz, the storage modulus of the gel was 4700 Pa. The gel was dominated by elasticity and had excellent mechanical properties.

## 4. Materials and Methods

### 4.1. Experimental Materials

The materials used in this work were acrylamide (AM), ammonium persulfate (APS), sodium chloride (NaCl), calcium chloride (CaCl_2_), tert-butanol hydrogen peroxide (TBHP), etc., which were all purchased from Shanghai Aladdin biochemical Technology Co., Ltd. in Shanghai, China. The gel used in the experiment was shear thixotropic polymer gel, which was independently developed by the laboratory. The water used in the experiment was deionized water, which was self-made in the laboratory. Lithium magnesium silicate was purchased from Bayer in Leverkusen, Germany. White oil was purchased from Shandong Chemical Co., Ltd. in Weifang, China. Barite was purchased from Zhejiang New Materials Co., Ltd. in Zhejiang, China.

### 4.2. Experimental Methods

#### 4.2.1. Gel Preparation

The preparation steps of the shear thixotropic polymer gelation are as follows: (a) Weigh a certain amount of the formation brine (91%), place it into a beaker and stir the brine using an electric mixer; (b) weigh a certain amount of the acrylamide (15.0%) and active polymer (2.0%), pour it into the brine slowly and stir the solution continuously to ensure that the polymer powder is dissolved uniformly; (c) weigh a certain amount of the crosslinking agent (BWL, 1.0 wt.%) and rheological regulator (lithium magnesium silicate, 4.0 wt.%), add it into the polymer solution prepared in step (b) slowly and stir the solution continuously to ensure that the crosslinking agent is dissolved uniformly; (d) weigh a certain amount of the toughening agent (lithium magnesium silicate, 1.5%), pour it into the solution prepared in step (c) slowly and stir the solution continuously to ensure that the toughening agent is dispersed uniformly; (e) weigh the initiator tert-butanol hydrogen peroxide (TBHP) with a concentration of 0.02%, add it into the solution prepared in step (d) slowly and stir the solution continuously to ensure that the tert-butanol hydrogen peroxide is dissolved uniformly. Then, the shear thixotropic polymer gelation can be achieved. The component content of the gel is shown in Table 3. The polymerization equation of acrylamide is shown in Formula (1).

(1) $$nCH_{2}=CHCONH_{2}\overset{Certain\;condition}{\to }{\left[CH_{2}=CHCONH_{2}\right]}_{n}$$

#### 4.2.2. Dilution Performance

In this work, water and white oil weighted with barite were used to replace deionized water to carry out anti-diluting experiments. The gelation properties of the gel system mixed with the two were investigated. The specific experimental methods were as follows:

(1) The shear thixotropic polymer gel system was prepared by weighting water with salt water instead of deionized water. The weighted shear thixotropic polymer gel system was diluted with salt water with a high concentration of sodium chloride, and the dispersion of the gel system and salt water was observed at different dilution levels. Dilution was carried out in the following four ways:
  (a) Pour 1/3 volume of the salt water into the gel system.
  (b) Pour 2/3 volume of the salt water into the gel system.
  (c) Pour 1/3 volume of the shear thixotropic polymer gel into the salt water.
  (d) Pour 2/3 volume of the shear thixotropic polymer gel into the salt water.
(2) In the dilution experiment of white oil, the method of mutual dilution between white oil and the gel system was used and the gel formation test was carried out. The dispersion of gel and white oil was observed at different dilution levels. The specific experimental steps were as follows:
  (a) The 90 mL gel system and 10 mL white oil were mixed and stirred, and placed in a measuring cylinder to observe the stratification state of the gel and white oil, respectively. The experimental recording time was 5 min, 10 min and 15 min, respectively. The gel solution was tested after being stirred evenly.
  (b) The 80 mL gel system and 20 mL white oil were mixed and stirred, and placed in a measuring cylinder to observe the stratification state of the gel and white oil, respectively. The experimental recording time was 5 min, 10 min and 15 min, respectively. The gel solution was tested after being stirred evenly.
  (c) The 70 mL gel system and 30 mL white oil were mixed and stirred, and placed in a measuring cylinder to observe the stratification state of the gel and white oil, respectively. The experimental recording time was 5 min, 10 min and 15 min, respectively. The gel solution was tested after being stirred evenly.

#### 4.2.3. Microstructure Characterization

The microstructure of the shear thixotropic polymer gel system was determined using a Quanta 200F environmental scanning electron microscope (ESEM, Carl Zeiss AG, Jena, Germany) [22]. First, a certain amount of the shear thixotropic gel sample was placed in liquid nitrogen for frozen sample preparation, and the microstructure of the shear thixotropic gel was fixed by rapid freezing. The frozen gel sample was then attached to the surface of a test copper sample stage using conductive tape, and we sprayed gold powder on the surface to enhance the conductivity of the gel sample. Finally, the sample was placed in the SEM sample chamber, and we set the magnification and other parameters according to requirements.

#### 4.2.4. Rheological Property

HAAKE Mars 60 (Thermo Fisher Scientific, Dreieich, Germany) was used to test the rheological properties of the shear thixotropic polymer gel system at different temperatures and salt ion concentrations. The type of conical rotor was C35 1°/Ti (the cone angle of the rotor was 1° and the diameter was 35 mm), and the gap between the rotor and the sample table was 0.053 mm during the test. The gel was cut into circular sample slices with a diameter of 30 mm and thickness of 3 mm. Among them, the test conditions of storage modulus (G′) and loss modulus (G″) was 1.0% constant strain, the range of vibration scanning frequency was 0–16 Hz and the range of angular frequency was 0.1–100 rad·s^−1^.

#### 4.2.5. Salt Resistance

The salt content of formation water had an important influence on the gelation of gel. Therefore, the shear thixotropic polymer gel system was prepared with 10,000 mg·L^−1^ and 100,000 mg·L^−1^ sodium chloride solution (NaCl) and 1000 mg·L^−1^ and 10,000 mg·L^−1^ calcium chloride solution (CaCl_2_), respectively, and the gelation tests were carried out by using tert-butyl alcohol hydrogen peroxide as initiator. The changes in morphology and structural stability of the final gel were observed and analyzed, and the salt resistance of shear thixotropic polymer gel was investigated.

#### 4.2.6. Temperature Resistance

In order to better understand the viscoelastic variation trend of the shear thixotropic polymer gel formula at different temperatures, the rheological properties of the gel at different temperatures were tested by using HAAKE Mars60 (Thermo Fisher Scientific, Dreieich, Germany). The test temperatures were 60 °C, 80 °C, 100 °C, 120 °C and 140 °C.

#### 4.2.7. Gelation Time

The high temperature initiator tert-butanol hydrogen peroxide was used to initiate the polymerization of gel system. Gelation time was tested by setting different initiator concentrations. The gelation state of the gel was observed by stirring with a glass rod. The effect of initiator concentrations of 0.1%, 0.2%, 0.3% and 0.5% on gelation time was studied at 140 °C.

#### 4.2.8. Tensile Strength

The mechanical properties of gel samples were tested by an electronic universal testing machine (CMT4000, Shenzhen new think material testing company, Shenzhen, China) at room temperature. Firstly, the undried gel was cut into cube pieces with a size of 2 mm × 2 mm × 12 mm, and the tensile rate was set at 50.0 mm/min. The tensile strength and elongation at break were measured and recorded by a clamp at both ends of the gel. The tensile strength was calculated using the following formula [62]:

(2) $$\sigma =\frac{F}{\left(l\cdot h\right)}$$

where σ is tensile strength, MPa. *F* is tension, N. *l* is sample width, cm. *h* is sample length, cm. *l* and *h* were both 2 cm.

## Figures and Tables

**Table 1:** Tensile test results of shear thixotropic polymer gel.

Serial Number	1	2	3
Tensile strength σ/MPa	4.46	4.21	3.66
Elongation ε/%	473	396	326

**Table 2:** The gelation time of gel under different initiator concentrations.

Temperature	Initiator Concentration	Gelation Time
140 °C	0.1%	6 h
	0.2%	5 h
	0.3%	4 h
	0.5%	2 h

**Table 3:** Component content of the gel.

Component	AM/%	Active Polymer/%	Initiator/%	Deionized Water/%	Rheological Regulator/%	Crosslinking Agent/%	Toughening Agent /%
Content	15	2	0.2	76.3	4	1	1.5
